# Identification of a Highly Potent Neutralizing Nanobody Against Human Adenovirus Type 4

**DOI:** 10.3390/vaccines13121192

**Published:** 2025-11-25

**Authors:** Tingting Yu, Wanrong Zhang, Peng Lv, Peijie Zhai, You Yang, Jianrong Wang, Zhengshan Chen, Guanying Zhang, Yunzhu Dong

**Affiliations:** 1 Academy of Military Medical Sciences, Beijing 100071, China; 2 College of Pharmacy, Nanjing University of Chinese Medicine, Nanjing 210023, China

**Keywords:** human adenovirus type 4, neutralizing antibody, nanobody, hexon, TRIM21

## Abstract

**Background:** Human adenovirus type 4 (HAdV-4), the sole member of species Human mastadenovirus E (HAdV-E), is of zoonotic origin and has established stable human transmission through recombination, conferring distinctive host adaptation and pathogenicity. It causes respiratory and ocular diseases, with a significant risk of severe pneumonia in children. No targeted antivirals are approved for routine use, leaving supportive care as the primary management. China bears a relatively high HAdV-4 disease burden in Asia. **Methods:** To generate neutralizing nanobodies (Nbs) against HAdV-4, we employed an alpaca immunization strategy using hexon protein from Ad4-RI67 strain, followed by the isolation of hexon-specific nanobodies. The epitope competition and molecular docking was employed to analysis the binding site of the Nbs’. We engineered VHH-Fc fusions by conjugating VHH domains to human IgG1 Fc. The lead candidate, NVA17, showed efficacy in both in vitro and in vivo (Stat1^+/−^ mouse model). Flow cytometric analysis was employed to assess the downstream immune effects of NVA17 in vivo. Its intracellular neutralization mechanism was further investigated through confocal microscopy by examining co-localization in TRIM21-overexpressing and knockdown cells. **Results:** The isolated nanobodies revealed epitopes distinct from those targeted by known antibodies. The lead candidate NVA17 demonstrated potent neutralizing activity in vitro (IC_50_ < 10 ng/mL). In the Stat1^+/−^ mouse model, NVA17 provided complete protection against lethal challenge, significantly reduced viral load in the lungs, and ameliorated pathological damage. NVA17 treatment dose-dependently reversed the virus-induced reduction in immune cell counts and enhanced cytotoxicity, suggesting a systemic immunomodulatory effect. Mechanistic studies indicated that the antiviral activity of NVA17 partly depends on the TRIM21-mediated antibody-dependent intracellular neutralization (ADIN) pathway, whereby TRIM21 terminates the viral life cycle by promoting viral degradation via K48-linked ubiquitination. **Conclusions:** We have identified multiple antibody candidates, particularly NVA17, with significant therapeutic potential for developing antibody-based treatments against HAdV-4. This offers a targeted intervention strategy to counter the current lack of specific antiviral therapies.

## 1. Introduction

Adenovirus, a non-enveloped double-stranded DNA virus [1], is a common pathogen whose infection can lead to a wide range of clinical manifestations, from mild colds to severe pneumonia [2]. It poses a heightened health risk to children and immunocompromised individuals [3], making it a significant public health concern. The virus exhibits distinct “population-specific” and “environment-dependent clustering” transmission patterns, with crowded settings or poorly ventilated enclosed environments being particularly conducive to clustered outbreaks. Among the numerous adenovirus serotypes, types 4 and 7 are the primary serotypes responsible for outbreaks of acute respiratory disease [4]. Specifically, HAdV-4 is classified within species Human mastadenovirus E, the currently only known member, characterized by a relatively unique genetic structure [5,6]. HAdV-4 infection typically presents as classic pharyngoconjunctival fever, characterized clinically by the triad of fever, pharyngitis, and acute conjunctivitis. Regarding prevention and control strategies, the primary measure relies on oral enteric vaccines [7]. However, there is still a lack of specific antiviral drugs for clinical treatment of adenovirus infection. Current management primarily involves supportive care to alleviate symptoms and cannot directly target the virus itself. This therapeutic gap not only affects patient recovery but also places an additional burden on healthcare systems [8]. Consequently, the development of safe and effective specific anti-adenovirus therapeutics has become an urgent priority in both research and clinical practice.

We had previously reported the development of a human monoclonal antibody (mAb) directed against human adenovirus type 4 (HAdV-4) and validated its potent neutralizing efficacy [9]. In parallel, nanobodies (Nbs)—a unique class of single-domain antibodies—have attracted increasing attention as promising anti-infective biologics, owing to their compact architecture and modular nature [10,11,12]. In contrast to conventional mAbs, nanobodies exhibit enhanced tissue permeability, facilitating efficient access to confined infection sites such as the respiratory mucosa [10]. By targeting cryptic and conserved viral epitopes, single-domain antibodies effectively counteract highly mutable pathogens, making nanobody screening a more suitable strategy [13]. Additionally, nanobodies demonstrate exceptional structural and thermal stability, favorable pharmacokinetics, and ease of engineering [10], rendering them compelling candidates for novel antiviral strategies.

Antibody-based therapeutics have demonstrated significant and unique advantages in the treatment of viral infections [14,15]. Antibody-based therapies play a central role in the treatment of viral infections by leveraging their high specificity for target engagement. The primary mechanism of action involves the precise recognition of key viral surface antigens by exogenously administered neutralizing antibodies. This binding directly blocks viral attachment to host cell receptors, thereby neutralizing viral infectivity [16]. Certainly, the therapeutic effect extends beyond direct viral neutralization to actively mobilizing and reprogramming immune cells within the body. Upon binding to viruses or infected cells via their Fab regions, therapeutic antibodies employ their Fc segment as a potent “recruitment signal,” rapidly activating natural killer (NK) cells of the innate immune system. This engagement significantly enhances the clearance of antibody-tagged viral particles or damaged cells. Notably, this process also shapes adaptive immunity through enhanced antigen presentation—viral particles opsonized by antibodies or debris from cleared infected cells can be more efficiently captured, processed, and presented by antigen-presenting cells such as dendritic cells. This process amplifies the activation and expansion of virus-specific CD8^+^ T lymphocytes, which in turn execute targeted elimination of infected host cells, thereby resolving established infections and establishing durable antiviral immunity [17].

TRIM21 (tripartite motif-containing protein 21) is a multifunctional intracellular protein that exhibits both cytosolic Fc receptor and E3 ubiquitin ligase activities, playing a central role in innate immune responses [18]. Conventionally, antibodies are thought to neutralize viruses extracellularly via their variable regions, thereby preventing viral entry into host cells [16]. Recent studies have revealed that antibodies against non-enveloped viruses can mediate a process known as antibody-dependent intracellular neutralization (ADIN): following the internalization of virus–antibody complexes into the cell, TRIM21 recognizes and binds to the complex, leading to viral degradation via the ubiquitin–proteasome pathway and activation of signaling cascades such as NF-κB and AP-1, thereby achieving efficient clearance of intracellular viruses [19]. As a typical non-enveloped virus, HAdV-4 has a cell membrane receptor that remains incompletely characterized [20]. Our previous work suggested that TRIM21 may serve as a potential intracellular receptor for the human monoclonal antibody 2CF4 against HAdV-4, although the underlying molecular mechanism requires further validation. In this study, we identified an engineered nanobody, NVA17, which is hypothesized to exert antiviral effects by activating TRIM21 and its downstream ubiquitin–proteasome pathway [21]. The present work aims to further elucidate the detailed molecular mechanism by which NVA17 mediates antiviral activity via the TRIM21-dependent ADIN pathway.

Therefore, this study employed an alpaca immunization strategy. Following three rounds of immunization with the hexon antigen protein, we successfully isolated multiple neutralizing nanobodies targeting HAdV-4 from the immune serum. To enhance its immune effector functions and drug-like properties [22], we further engineered it by fusion with a human Fc fragment. In in vitro assays, the lead candidate, designated NVA17, exhibited excellent neutralizing activity. In a STAT1-deficient mouse model of lethal HAdV-4 infection [9], this antibody provided complete protection and effectively modulated systemic immune responses.

## 2. Materials and Methods

### 2.1. Viruses and Cell Lines

The human embryonic kidney cell line (HEK293T) and the human lung adenocarcinoma epithelial cell line (A549) were cultured in Dulbecco’s Modified Eagle Medium (DMEM) (Gibco, Grand Island, NE, USA, Cat. No. C11995500BT) supplemented with 10% fetal bovine serum, 100 μg/mL streptomycin, and 100 IU/mL penicillin (Gibco, Cat. No. 15140122). All cells were maintained at 37 °C in a humidified incubator with 5% CO_2_. Expi293F human cells (derived from the 293F cell line) were grown in suspension using Expi293 Expression Medium at 37 °C, 5% CO_2_, and 110 rpm on an orbital shaker. The recombinant adenoviruses Ad4-RI67 and Ad4-Luc used in this study were either maintained or generated in our laboratory.

### 2.2. Expression and Purification of the Hexon Protein

To obtain the HAdV-4 viral protein, infected 293F cells were cultured for 72 h and then subjected to three freeze–thaw cycles for complete lysis. The lysate was centrifuged at 4 °C and 4500 rpm for 15 min. The supernatant was collected and filtered through a 0.22 μm filter (PALL, Cat. No. 4612). The filtrate was first purified using a Core 700 size-exclusion column (Cytiva, Uppsala, Sweden, Cat. No. 17548115) equilibrated with a buffer containing 20 mM Tris and 150 mM NaCl (pH 7.5). The elution peak corresponding to the target protein, monitored by UV absorbance, was collected. This fraction was concentrated using a 30 kDa ultrafiltration tube (Merck Millipore, Carrigtwohill, Co Cork, Ireland, Cat. No. UFC903096) and then further purified by a Superdex 200 size-exclusion column (Cytiva) using PBS as the mobile phase. Again, the elution peak of the target protein was collected based on UV monitoring. The purified fractions were pooled, and the protein concentration was determined using a BCA assay kit (Merck Millipore, Darmstadt, Germany, Cat. No. 23225). The final protein samples were aliquoted and stored at −80 °C. The purity and immunoreactivity of the protein samples were verified by SDS-PAGE and Western blotting, respectively.

### 2.3. Alpaca Immunization

To isolate neutralizing Nbs against HAdV-4, an alpaca was subcutaneously immunized with 1 mg of recombinant HAdV-4 hexon protein formulated in Freund’s adjuvant on day 0. This was followed by two booster immunizations administered at two-week intervals, each with 0.7 mg of hexon protein in Freund’s adjuvant, resulting in a total of three immunizations. Beginning after the second immunization, blood was collected 7 days after each booster, and serum antibody titers were measured by ELISA. Whole RNA was extracted from peripheral blood mononuclear cells and reverse-transcribed into cDNA after quality verification by electrophoresis. The VHH-encoding sequences were amplified by two rounds of PCR, purified, digested with SfiI, and cloned into the phagemid vector pComb3X. The ligated product was electroporated into XL1-Blue competent cells, yielding an initial library with a capacity of 1.2 × 10^9^ CFU. Random clone sequencing confirmed 100% correctness. Finally, the library was infected with VCSM13 helper phage to generate the nanobody phage display library.

### 2.4. VHH Library Generation

To screen for specific nanobodies targeting human adenovirus type 4 (HAdV-4), two rounds of biopanning were performed. The high-binding plates were coated with HAdV-4 antigen (5 μg/mL) and incubated overnight at 4 °C, followed by blocking with 3% skim milk powder to reduce non-specific binding. The phage display library, with an initial capacity of 1.2 × 10^9^ CFU (approximately 5 × 10^12^ phage particles), was added to the antigen-coated wells and incubated at 37 °C for 2 h to facilitate binding. Unbound and weakly bound phages were removed by washing with PBS containing 0.1% Tween 20 (PBST). To increase selection stringency, the number of washing steps was incrementally raised—10 times in the first round and 15 times in the second round. Specifically bound phages were eluted using 0.1 M glycine-HCl (pH 2.2) and immediately neutralized with 1 M Tris-HCl (pH 7.4). The eluted phages were used to infect E. coliXL1-Blue cells, rescued with VCSM13 helper phage, amplified, and purified by PEG 8000/NaCl precipitation for the subsequent round of panning. Enrichment efficiency was evaluated after each round by phage titer determination. Following the second round of panning, individual clones were selected for phage ELISA screening. An HRP-conjugated anti-M13 antibody was used as the secondary antibody. Clones exhibiting an OD_450_ value ≥ 0.5 and a positive-to-negative (P/N) ratio greater than 3 were identified as positive binders. Positive candidates were sent to Sangon Biotech (Shanghai, China) for sequencing and sequence alignment. In all ELISA, 5% bovine serum albumin (BSA) was used as the negative control.

### 2.5. Protein Purification

To obtain VHH-Fc fusion proteins, the VHH coding sequences from selected phage clones were amplified and cloned into the pcDNA3.4 eukaryotic expression vector. The vector contains a CMV promoter, a tPA signal peptide, and the human IgG1 Fc gene frame, allowing in-frame fusion expression under the control of the SV40 poly(A) signal when the gene of interest is inserted into the multiple cloning site. The recombinant plasmids were transfected into Expi293F cells using ExpiFectamine 293 Transfection Kit (Thermo Fisher Scientific, Carlsbad, CA, USA, Cat. No. A14526), followed by 4 days of culture after which the supernatant was collected. The collected supernatant was pre-treated by two-step centrifugation (800× *g* for 10 min and 4000× *g* for 15 min) and filtration through a 0.22 μm membrane (PALL, Bristol, UK, Ca. No. 4612). The target protein was then purified using an ÄKTA pure 150 system with a 5 mL Protein A affinity chromatography column, equilibrated with PBS (pH 7.5) and eluted with 0.1 M glycine (pH 2.7). Protein purity was analyzed by SDS-PAGE, and concentration was determined by either the BCA method or UV absorbance measurement.

### 2.6. Enzyme-Linked Immunosorbent Assay (ELISA)

To evaluate the antibody binding activity to hexon protein, an indirect ELISA was performed. Purified hexon protein was coated onto 96-well plates at 2 μg/mL (100 μL/well) and incubated overnight at 4 °C. After discarding the coating solution, the plates were washed three times with PBS containing 0.02% Tween 20 (PBST). Each well was then blocked with 100 μL of PBS containing 2% BSA at 37 °C for 1 h. Subsequently, purified antibodies were subjected to threefold serial dilution starting from 1 μg/mL (with three replicates per concentration). A volume of 100 μL of diluted antibody was added to each well and incubated at 37 °C for 1 h, followed by three washes with PBST. Then, HRP-conjugated goat anti-human IgG Fc secondary antibody (Abcam97225, Cambridge, UK, 1:10,000 dilution) diluted at 1:10,000 was added and incubated at 37 °C for 1 h in the dark. After washing, 100 μL of TMB substrate (Solarbio, Beijing, China, Cat. No. PR1200) was added to each well and incubated at room temperature in the dark for 5–6 min. The reaction was terminated by adding 50 μL of stop solution. The absorbance was measured at 450 nm with a reference wavelength of 630 nm using a microplate reader, and the specific binding signal was calculated after background correction.

### 2.7. Nanobodies Neutralization Experiment Against HAdV-4

The Nbs were serially diluted threefold in DMEM supplemented with 10% FBS, starting from an initial concentration of 50 μg/mL. An equal volume (50 μL) of the diluted Nbs was mixed with 50 μL of the Ad4-Luc recombinant virus solution and incubated (37 °C, 5% CO_2_) for 1 h. Subsequently, 100 μL of an A549 cell suspension (2 × 10^5^ cells/mL) was added to each well. (final volume: 200 μL/well), with appropriate controls included (positive control: virus plus cells; negative control: cells only). After 24 h of culture at 37 °C under 5% CO_2_, the medium was aspirated, and cells were lysed with luciferase cell culture lysis reagent for 10 min. Luciferase activity was measured using a 20 μL aliquot of the lysate. The neutralization efficiency was calculated based on the signal reduction in test groups relative to the virus control.

To investigate the role of TRIM21 in antibody-mediated intracellular neutralization, the same assay was performed using TRIM21-knockdown, TRIM21-overexpressing A549 cells, and their corresponding empty vector controls instead of wild-type cells. The neutralizing efficiencies across these genetically modified cell lines were compared.

To examine the dependency of the neutralization process on the proteasomal pathway, the proteasome inhibitor MG132 (1 μM) was added to the culture wells at the time of seeding with either wild-type or TRIM21-overexpressing A549 cells. Following a 24 h incubation at 37 °C, luciferase activity was measured and compared with that of untreated control groups.

### 2.8. Authentic Virus Neutralization Assay

The neutralizing activity against wild-type HAdV-4 (strain RI67) was determined using a microneutralization assay under Biosafety Level 2 (BSL-2) conditions. Briefly, A549 cells were seeded in 96-well plates at a density of 5 × 10^4^ cells/well in 100 μL of DMEM supplemented with 10% FBS and cultured overnight at 37 °C under 5% CO_2_. Antibodies were subjected to threefold serial dilution starting from 50 μg/mL and mixed with an equal volume of HAdV-4 containing 100 TCID_50_, followed by incubation at 37 °C for 1 h. After removing the culture medium, 100 μL of the virus-antibody mixture was added to the cells and incubated for 3 days at 37 °C with 5% CO_2_. Cytopathic effects were examined by microscopy, and cell viability was quantified using a CCK-8 assay. For the CCK-8 assay, the supernatant was discarded, and 100 μL of DMEM containing 2% FBS and 4% CCK-8 reagent was added to each well, followed by incubation at 37 °C for 40 min in the dark. Absorbance was measured at 450 nm using a microplate reader. The half-maximal inhibitory concentration (IC_50_) was calculated based on antibody dilution concentrations and corresponding cell viability rates.

### 2.9. Competition-Binding ELISA

A competitive ELISA was performed to analyze the binding competition of antibodies to the hexon protein. Antibodies (100 μg) were biotinylated using EZ-Link™ Sulfo-NHS-Biotin at a 1:20 molar ratio (antibody:biotin), purified using a desalting column, and stored at 4 °C in the dark after concentration measurement. Ninety-six-well plates were coated with 1 μg/mL hexon protein (100 μL/well) and incubated overnight at 4 °C. After washing with PBST, the plates were blocked with 2% BSA for 1 h at 37 °C. A total of 50 μL of unlabeled competing antibody (100 × EC_50_) was added to each well and incubated for 30 min at 37 °C, followed by the addition of 50 μL of biotinylated detection antibody (1 × EC_50_) and another 30 min incubation at 37 °C. After washing, a 1:10,000 dilution of streptavidin-HRP was added and incubated for 1 h at 37 °C. The reaction was developed with TMB substrate for 6 min, stopped, and the absorbance was measured. The competition rate was calculated as (OD of competition well/OD of control well) × 100%. A competition rate of <33.3% was considered strong competition, 33.3–66.7% as weak competition, and >66.7% as no competition.

### 2.10. Surface Plasmon Resonance (SPR) Assay

Antibody–antigen binding kinetics were determined by surface plasmon resonance (SPR) using a Biacore T200 instrument (Cytiva). The antibody was diluted in HBS-EP^+^ buffer (Cytiva) to a concentration of 0.5 μg/mL and captured on a Protein A sensor chip at a flow rate of 10 μL/min for 60 s. The purified antigen was tested at serially diluted concentrations (100, 50, 25, 12.5, and 6.25 nM) at a flow rate of 30 μL/min. The association and dissociation phases were monitored for 120 s and 900 s, respectively.

### 2.11. In Vivo Animal Challenge Experiment

All procedures involving 6- to 8-week-old male STAT1^+/−^ transgenic mice (Shanghai Model Organisms Center, Beijing, China), which were housed under BSL-2 conditions with ad libitum access to food and water, were approved by the Animal Ethics Committee of the Laboratory Animal Center, Academy of Military Medical Sciences. In the prophylactic model, mice were intraperitoneally administered 100 μL of PBS containing graded doses (50, 10, or 2 μg) of nanobody NVA17. 24 h later, they were challenged intraperitoneally with 6 × 10^10^ PFU of authentic HAdV-4. In the therapeutic model, mice were first infected with an equivalent viral dose and then received the same nanobody treatments at 3 h post-infection. Survival rates and body weight changes were monitored daily for 14 days post-infection (*n* = 4). For the viral load experiment, on day 3 post-infection, euthanasia was performed via cervical dislocation (*n* = 4). Heart, liver, spleen, lung, and kidney tissues were collected for viral load quantification by reverse transcription-quantitative polymerase chain reaction (RT-qPCR). Histopathological examination was conducted after 48 h of fixation in 10% formalin, paraffin embedding, sectioning, and hematoxylin-eosin (H&E) staining.

### 2.12. Viral Load Quantification by RT-qPCR

Viral load was quantified by quantitative real-time PCR (RT-qPCR), using DNA from viral stocks as a standard to calculate genomic DNA copy numbers. Genomic DNA was extracted from tissue samples using the QIAamp DNA Mini Kit (Qiagen, Hilden, Germany, Cat. No. 57704). Amplification was performed using SYBR Green Supermix with the following primers: HAdV-4-F (5′-CAAGGACTACCAGGCCGTCA-3′) and HAdV-4-R (5′-GTTAGCATAGAGCATGTTCT-3′). The thermal cycling protocol included an initial step at 50 °C for 2 min and 95 °C for 10 min, followed by 40 cycles of denaturation at 95 °C for 15 s and annealing/extension at 60 °C for 1 min. A melting curve analysis was performed by heating from 60 °C to 95 °C at a rate of 0.15 °C/s with continuous fluorescence acquisition to verify amplification specificity. Viral genome copies were quantified using a standard curve generated from serially diluted DNA standards.

### 2.13. Flow Cytometry

Spleens were aseptically harvested from euthanized mice and placed in ice-cold PBS containing 2% FBS. A single-cell suspension was obtained by gently homogenizing the spleen through a 70 μm cell strainer. The filtrate was centrifuged at 600× *g* for 5 min at 4 °C, and the supernatant was discarded. The cell pellet was treated with red blood cell lysis buffer for 1–2 min at room temperature protected from light. After stopping the reaction, the sample was centrifuged again under the same conditions. Cell viability and concentration were assessed using an automated cell counter. Cells were adjusted to a concentration of 1 × 10^7^ cells/mL and aliquoted into a U-bottom 96-well plate. Sequential incubations were performed as follows: Fc receptor blocking with anti-mouse CD16/CD32 antibody (20 min, 4 °C, dark); staining with a fixable viability dye (eFluor™ 506; 20 min, 4 °C, dark); and incubation with a pre-titrated cocktail of fluorochrome-conjugated antibodies (30 min, 4 °C, dark). Cells were then washed three times with PBS containing 2% FBS by centrifugation. Finally, the cell suspension was filtered through a 40 μm cell strainer prior to flow cytometry acquisition.

### 2.14. Simulation of Structure of Antigen–Antibody Complexes Through Alphafold3

The amino acid sequences of the adenovirus hexon protein (obtained from the GenBank database) and the nanobody heavy chain were input into a locally deployed AlphaFold3 (version 3.0.0) platform for complex structure prediction. Simulations were performed using default parameters. The generated models were evaluated based on multiple confidence metrics, including the predicted local distance difference test (pLDDT), predicted aligned error (PAE), predicted template modeling (pTM) score, and interface pTM (ipTM) score. The highest-ranking model was selected and visualized using UCSF ChimeraX (version 1.8) to analyze key interacting residues at the antigen–antibody interface.

### 2.15. Immunofluorescence Assay

Wild-type, TRIM21-overexpressing, and TRIM21-knockdown A549 cells were seeded on coverslips in 24-well plates at a density of 5 × 10^4^ cells/well and cultured overnight at 37 °C for adhesion. Prior to infection, cells were gently washed twice with DMEM. Ad4-RI67 virus (1 × 10^8^ PFU) was mixed with 10 μg of nanobody NVA17 (negative control: non-neutralizing nanobody 127) in 500 μL of DMEM and pre-incubated at room temperature for 30 min. After removing the culture medium, 500 μL of the virus-antibody mixture was added to each well and incubated at 37 °C for 6 h. Post-infection, cells were washed three times with PBS, fixed with 4% paraformaldehyde for 20 min at room temperature, permeabilized with 0.5% Triton X-100 for 15 min, and blocked with PBS containing 5% BSA and 0.1% Tween-20 for 1 h. For immunostaining, cells were incubated with a rabbit anti-TRIM21 primary antibody (1:200 dilution) at 4 °C for 1 h, followed by three washes with PBST. Subsequently, cells were incubated with Alexa Fluor 594-conjugated goat anti-rabbit secondary antibody (1:400) and DyLight 488-conjugated goat anti-human secondary antibody (1:1000) for 30 min each at room temperature in the dark, with three PBST washes after each secondary antibody incubation. Nuclei were stained with DAPI (1:2000 dilution) for 10–15 min. All fluorescence images were acquired using a Zeiss Axio Observer microscope (Carl Zeiss Microscopy GmbH, Jena, Germany) equipped with a 63× objective.

### 2.16. Differential Scanning Calorimetry (DSC) Assay

The thermodynamic stability of the antibody was assessed by differential scanning calorimetry (DSC) using a Nano DSC instrument. The system was equilibrated for 1 h using the DSCRun™ software (version 3.11.0) prior to measurement. The antibody sample was diluted to 1 mg/mL in PBS, and the reference (PBS) was degassed for 10 min using a degassing station. After pressurizing the system to 45 psi, the temperature program was initiated with a heating rate of 1 °C/min from 25 °C to 100 °C, with an initial equilibration time of 600 s. Multiple replicate scans were performed to verify the reproducibility of the results and the stability of the system.

### 2.17. Cytotoxicity Assay of MG132

A549 cells were seeded in 96-well plates at a density of 8 × 10^4^ cells/well in 100 μL of DMEM supplemented with 10% FBS and cultured overnight at 37 °C under 5% CO_2_. After removing the medium, cells were treated with 100 μL of serially diluted MG132 in DMEM containing 10% FBS and incubated for 24 h. Following microscopic examination for cytopathic effect, cell viability was assessed using a CCK-8 assay. Briefly, the supernatant was discarded, and cells in each well were incubated with 100 μL of DMEM containing 2% FBS and 4% CCK-8 reagent at 37 °C in the dark for 40 min. Absorbance was measured at 450 nm using a microplate reader.

### 2.18. Quantification and Statistical Analysis

The half-maximal effective concentration (EC_50_) of sera and neutralizing antibodies in the ELISA was determined by four-parameter nonlinear regression analysis using GraphPad Prism software (version 10.2.1). The neutralization percentage in the viral neutralization assay was calculated as follows: (sample signal − blank control signal)/(virus control signal − blank control signal) × 100%, and the data were fitted using a three-parameter nonlinear regression model in GraphPad Prism (v10.2.1). A phylogenetic tree was constructed with ChiPlot, and the complementarity-determining regions (CDR1, CDR2, and CDR3) of nanobodies were aligned using Abalign (version 1.2.9). Affinity parameters derived from surface plasmon resonance (SPR) experiments—including the association rate (Ka), dissociation rate (Kd), and equilibrium dissociation constant (K_D_)—were calculated using a 1:1 binding model in the Biacore T200 Evaluation Software (version 3.2). For statistical analysis, a two-tailed Student’s *t*-test was used for comparisons between two groups, and one-way analysis of variance (ANOVA) followed by Dunnett’s test was applied for comparisons among multiple groups. Data are expressed as the mean ± standard deviation, with significance levels set at * *p* < 0.05, ** *p* < 0.01, and *** *p* < 0.001.

## 3. Results

### 3.1. Screening of Nanobodies Targeting the Hexon Protein of Human Adenovirus Type 4 (HAdV-4)

**Figure 1:**
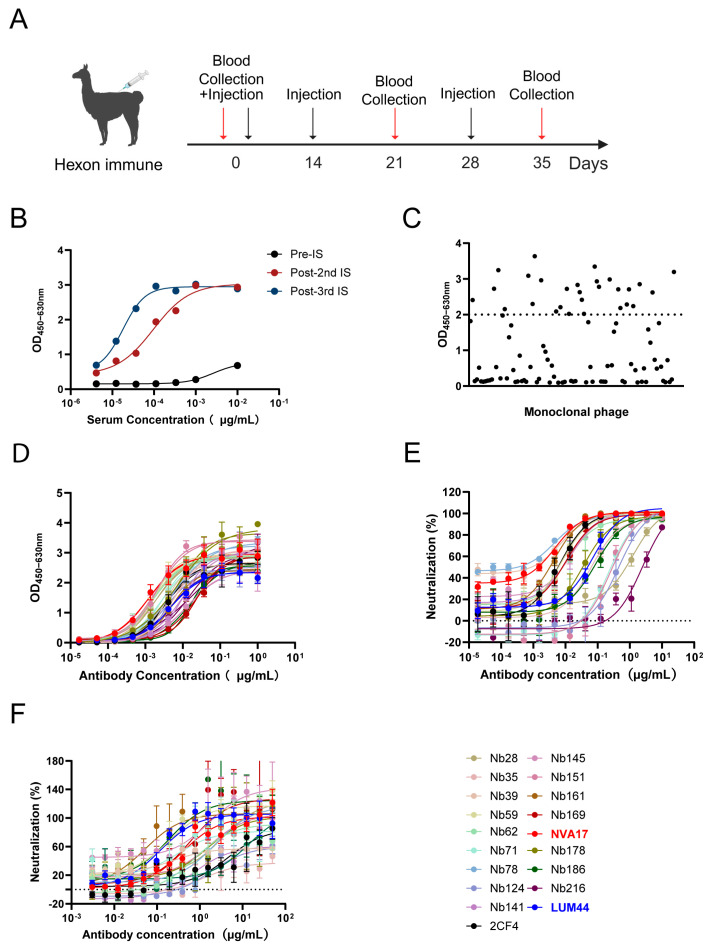
Screening and functional characterization of HAdV-4-specific nanobodies. (**A**) Schematic timeline of the alpaca immunization protocol. (**B**) Serum antibody titers against HAdV-4 hexon protein measured by ELISA before immunization (Pre-IS), after the second (Post-2nd IS), and after the third immunization (Post-3rd IS). (**C**) ELISA-based identification of monoclonal phages binding to hexon; data points represent individual clones (*n* = 96), with clones showing OD_450–630_ > 2 considered positive. (**D**) Binding curves of purified nanobodies to HAdV-4 hexon protein. (**E**) Neutralization activity of hexon-reactive nanobodies against HAdV-4-Luc reporter virus in A549 cells. (**F**) Neutralization activity against authentic HAdV-4 virus in A549 cells. Data in (**D**–**F**) are presented as the mean ± SD from three replicates in one representative experiment and are representative of three independent biological replicates.

For the isolation of HAdV-4-targeting nanobodies (Nbs), we employed the viral hexon protein as an immunogen. The protein was purified from 293F cells infected with the laboratory-preserved Ad4-RI67 strain and used for multiple immunization rounds (Figure S1A). An alpaca was immunized three times at two-week intervals (Figure 1A). Serological analysis showed a half-maximal inhibitory concentration (IC_50_) below 55 ng/mL against HAdV-4 after the final immunization (Figure 1B), indicating the induction of a high-potency humoral immune response.

Peripheral blood mononuclear cells (PBMCs) were isolated and total RNA was extracted. VHH gene fragments were amplified by a two-step nested PCR and cloned to construct a phage display library with a final titer of 4 × 10^12^ CFU/mL (Figure S1B). After two rounds of biopanning, 96 individual clones were selected from the enriched library for phage ELISA screening (Figure 1C), leading to the identification of multiple antigen-binding Nbs (Figure S1B). For functional analysis, selected VHH sequences were fused to a human IgG1 Fc domain and transiently expressed in Expi293F cells to produce VHH-Fc fusion proteins for downstream characterization.

To functionally characterize the candidate nanobodies, we systematically evaluated their binding affinity and neutralizing potency. ELISA screening identified 18 Nbs exhibiting strong binding to the hexon protein, with half-maximal effective concentration (EC_50_) values ranging from 1.2 to 24 ng/mL (Figure 1D), indicating high-affinity interactions with HAdV-4 hexon. Neutralization capacity was initially assessed using a replication-competent Ad4-Luc recombinant virus, in which the E3 region was replaced with a luciferase reporter gene. All 18 Nbs effectively inhibited Ad4-Luc infection, with seven clones demonstrating particularly potent neutralization (IC_50_ < 10 ng/mL) (Figure 1E). To further validate the activity against authentic virus, a neutralization assay was performed in A549 cells. The high consistency of its results with the pseudovirus data (Figure 1F) underscores the potent anti-HAdV-4 efficacy of the selected Nbs.

### 3.2. Characterization of Hexon Protein-Specific Humanized Nanobodies

**Figure 2:**
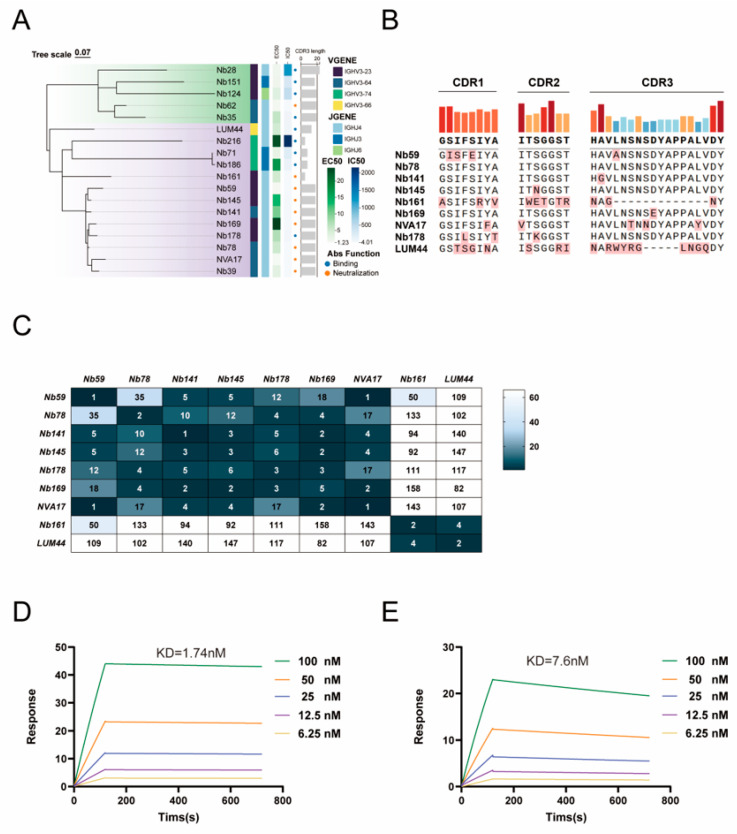
(**A**) Phylogenetic tree showing the relationships among nanobody sequences (generated using ChiPlot). (**B**) Alignment result showing the sequence conservation in the CDRs (CDR1, CDR2, CDR3) of selected nanobodies (performed with Abalign). Key divergent residues are highlighted in pink. (**C**) Competition binding profile of selected nanobodies (Nbs). The values indicate the percentage inhibition of binding for the competing nanobody relative to the primary antibody. Dark blue boxes (white numbers): Nbs with >66% inhibition are considered competitive binders. White boxes (black numbers): Nbs with <33% inhibition are non-competitive. Boxes with an intermediate phenotype (33–66% inhibition) are shaded in light blue. (**D**,**E**) Surface plasmon resonance (SPR) sensorgrams and binding kinetics for the interaction of (**D**) NVA17 and (**E**) LUM44 with HAdV-4 hexon protein. The derived kinetic parameters are as follows. NVA17 (**D**): KD = 1.742 nM, ka = 2.536 × 10^4^ M^−1^s^−1^, kd = 4.418 × 10^−5^ s^−1^. LUM44 (**E**): KD = 7.599 nM, ka = 4.656 × 10^4^ M^−1^s^−1^, kd = 3.539 × 10^−4^ s^−1^.

To elucidate the relationship between antibody genotype and functional phenotype, we constructed a phylogenetic tree based on the amino acid sequences of the variable heavy chain (VH). The resulting phylogeny revealed a strong correlation between antibody function and germline gene usage: neutralizing nanobodies, binding nanobodies, and non-binding nanobodies clustered into distinct phylogenetic clades, indicating that functional properties are closely associated with specific VH gene lineages (Figure 2A).

Based on the phylogenetic analysis, we further compared the amino acid sequences of the complementarity-determining regions (CDRs) from nine representative nanobodies. The results revealed that compared to the other nanobodies, Nb161, NVA17, and LUM44 exhibited distinct CDR lengths and key residue compositions in CDR1, CDR2, and CDR3 (Figure 2B). To evaluate potential epitope, overlap among these nanobodies, we subsequently performed pairwise competition binding assays using an ELISA-based approach. The assays displayed distinct competition patterns, with multiple nanobody pairs showing mutually exclusive binding characteristics; notably, no significant competition was observed between NVA17 and LUM44, suggesting that they recognize distinct epitopes (Figure 2C). These findings demonstrate that the nanobody response induced by HAdV-4 exhibits considerable diversity in epitope recognition on the hexon protein.

Building on epitope competition data indicating that NVA17 and LUM44 recognize distinct antigenic sites, we quantitatively characterized their binding kinetics with the hexon protein using surface plasmon resonance (SPR). SPR analysis demonstrated high-affinity binding for both nanobodies, with equilibrium dissociation constants (K_D_) of 1.74 nM for NVA17 and 7.6 nM for LUM44 (Figure 2D,E). Furthermore, differential scanning calorimetry (DSC) analysis revealed that both nanobodies exhibited high thermal stability, with melting temperatures (T_m_) exceeding 65 °C (Figure S1C,D), underscoring their biophysical robustness and therapeutic potential. To assess neutralization breadth, NVA17 and LUM44 were tested against luciferase-expressing HAdV-5 (Ad5-Luc) and HAdV-7 (Ad7-Luc). Neither antibody exhibited cross-neutralizing activity (Figure S1E,F), demonstrating serotype-specific activity restricted to HAdV-4.

### 3.3. Structural Prediction and Functional Validation of Key Binding Residues in Nanobodies

**Figure 3:**
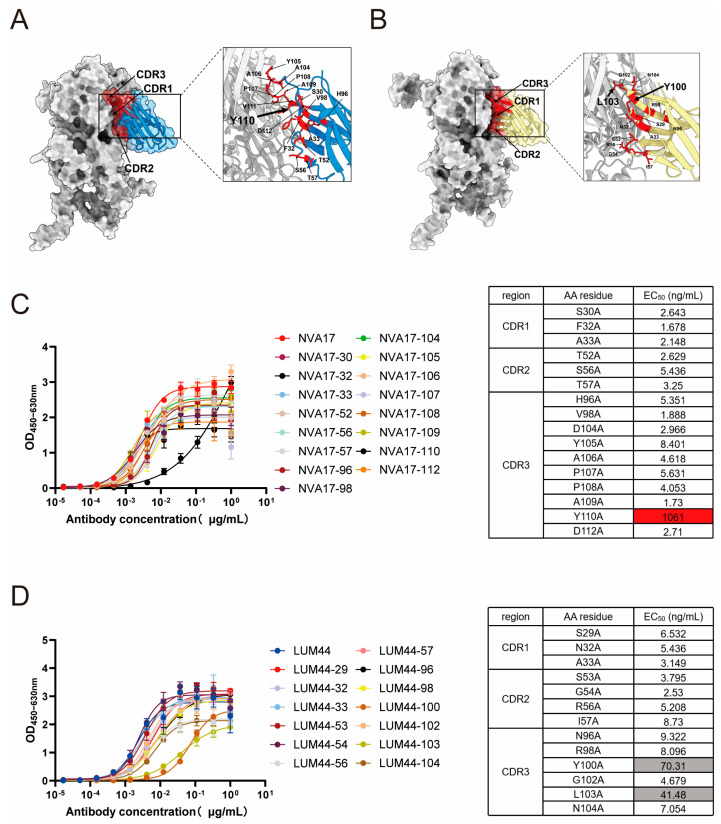
Validation of key residues at the hexon–NVA17 and hexon–LUM44 interfaces. (**A**,**B**) Predicted complex structures of (**A**) NVA17 and (**B**) LUM44 bound to HAdV-4 hexon, generated by AlphaFold-based molecular docking. (**C**) Analysis of binding activity of NVA17 heavy-chain mutants to hexon: (**left**) ELISA measuring binding activity relative to wild-type NVA17 (WT-NVA17); (**right**) heatmap of binding affinity (EC_50_) color-coded as white (high affinity, EC_50_ < 10 nM) and red (no detectable binding). (**D**) Binding activity of LUM44 heavy-chain mutants to hexon measured by ELISA and normalized to wild-type LUM44 (WT-LUM44). heatmap of binding affinity (EC_50_) color-coded as white (high affinity, EC_50_ < 10 nM) and gray (low affinity, 10 nM ≤ EC_50_ ≤ 100 nM). All data are presented as the mean ± SD from three replicates in one representative experiment and are representative of three independent biological experiments.

To elucidate the molecular basis of nanobody–hexon recognition, we predicted the three-dimensional structures of NVA17 and LUM44 in complex with the antigen using AlphaFold3-multimer. The resulting models exhibited strong concordance with experimental structural data (Figure 3A,B), offering high-resolution insight into the binding interfaces. Guided by these predictions, we performed systematic alanine scanning mutagenesis, targeting 16 paratope residues in NVA17 and 13 in LUM44 across their CDR loops. ELISA-based binding assessment revealed that substitution of TRP110 in the HCDR3 of NVA17, as well as TYR100 and LEU103 in LUM44, markedly impaired antigen recognition (Figure 3C,D), highlighting the central role of HCDR3 in epitope engagement. Notably, the NVA17 W110A mutant exhibited an approximately 1000-fold decrease in binding affinity, as reflected by a sharp increase in half-maximal effective concentration (EC_50_), identifying this residue as a structural linchpin for high-affinity binding. Based on its superior binding affinity, neutralization potency, and recombinant expression yield, NVA17 was prioritized for subsequent mechanistic studies.

### 3.4. Prophylactic and Therapeutic Efficacy of NVA17 in STAT1^+/−^ Mouse Models

**Figure 4:**
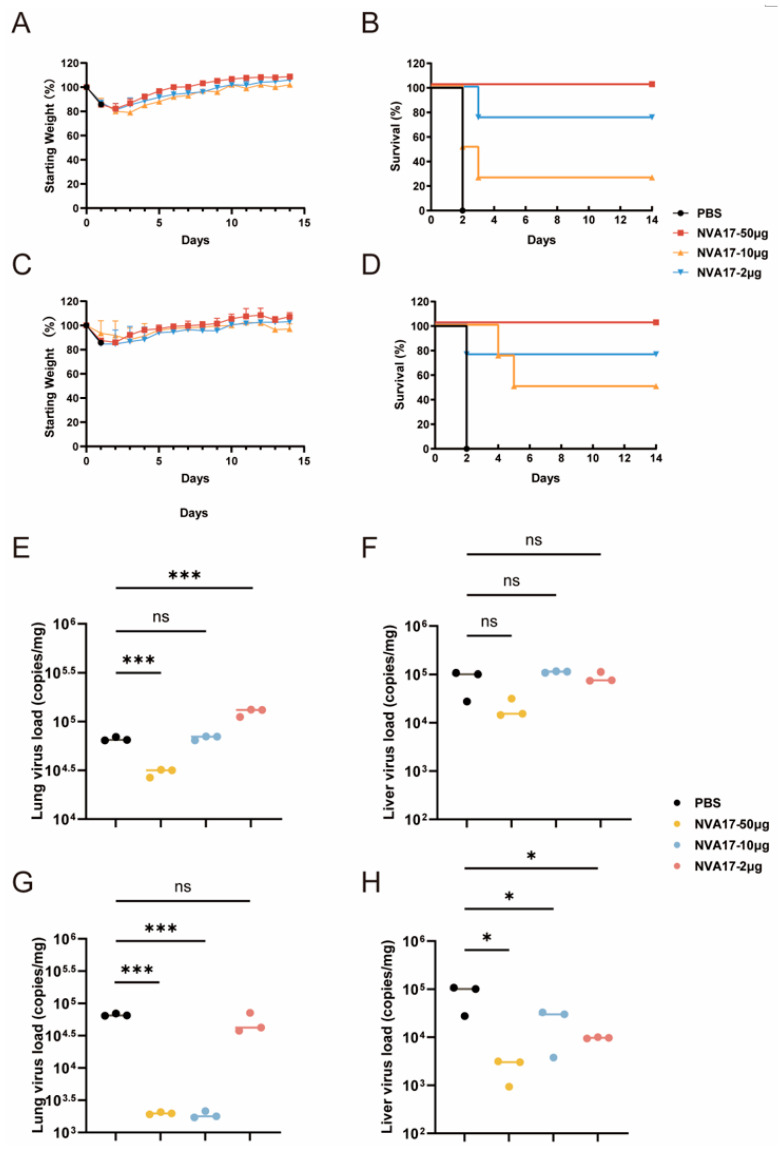
In vivo prophylactic and therapeutic efficacy of NVA17 (2 μg–50 μg) against HAdV-4 in STAT1^+/−^ mice. (**A**,**C**) Body weight changes in the (**A**) prophylactic and (**C**) therapeutic treatment groups. Values represent mean ± SD (*n* = 4). Mice in the virus control group received PBS at the same volume as the antibody. (**B**,**D**) Survival rates in the (**B**) prophylactic and (**D**) therapeutic treatment groups (*n* = 4). (**E**,**F**) Viral loads measured in the (**E**) lungs and (**F**) liver on day 3 post-infection from the prophylactic group (*n* = 3). (**G**,**H**) Viral loads measured in the (**G**) lungs and (**H**) liver on day 3 post-infection from the therapeutic group (*n* = 3). Statistical significance was determined by one-way ANOVA with Dunnett’s test (* *p* < 0.05, *** *p* < 0.001, ns (not significant) *p* > 0.05.).

Given the superior neutralizing activity of nanobody NVA17 compared to LUM44, it was selected for further in vivo functional evaluation. Using an established lethal HAdV-4 infection model in STAT1^+/−^ transgenic mice, we systematically assessed the protective efficacy of NVA17. STAT1^+/−^ mice were inoculated with 6 × 10^10^ PFU of authentic HAdV-4 (Figure S2A). All mice in the virus-infected control group succumbed within 2 days post-infection, exhibiting pronounced weight loss. In contrast, prophylactic administration of NVA17 at 50 μg (∼2.5 mg/kg), 10 μg (∼0.5 mg/kg), or 2 μg (∼0.1 mg/kg) one day prior to challenge significantly improved survival, with surviving animals displaying transient weight loss followed by steady recovery (Figure 4A,B). Similarly, therapeutic intervention with the same doses at 3 h post-infection enhanced survival rates and promoted weight restoration (Figure 4C,D). Notably, the 50 μg dose conferred complete protection in both prophylactic and therapeutic regimens. Quantitative PCR revealed substantial reductions in viral loads in lung and liver tissues under both treatment regimens (Figure 4E–H), accompanied by markedly alleviated pulmonary pathology (Figure S2B). In contrast, only mild pathological changes were observed in the heart and kidneys across all groups, with no significant intergroup differences (Figure S2B), suggesting that these organs are not primary targets of HAdV-4 infection. These in vivo results demonstrate that nanobody NVA17 exhibits a clear dose-dependent protective effect, with low to medium doses (2 μg–10 μg) providing partial protection and a high dose (50 μg) conferring complete protection, underscoring its strong potential for both prophylaxis and therapy against HAdV-4 infection.

### 3.5. Identification of Immune Response Following Nb177 Treatment

**Figure 5:**
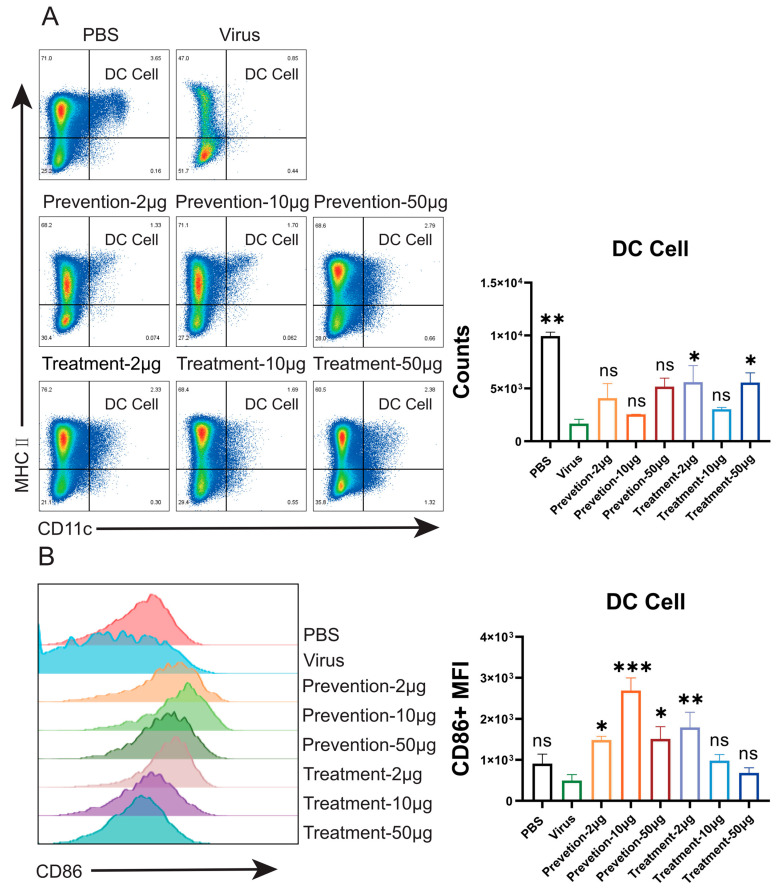

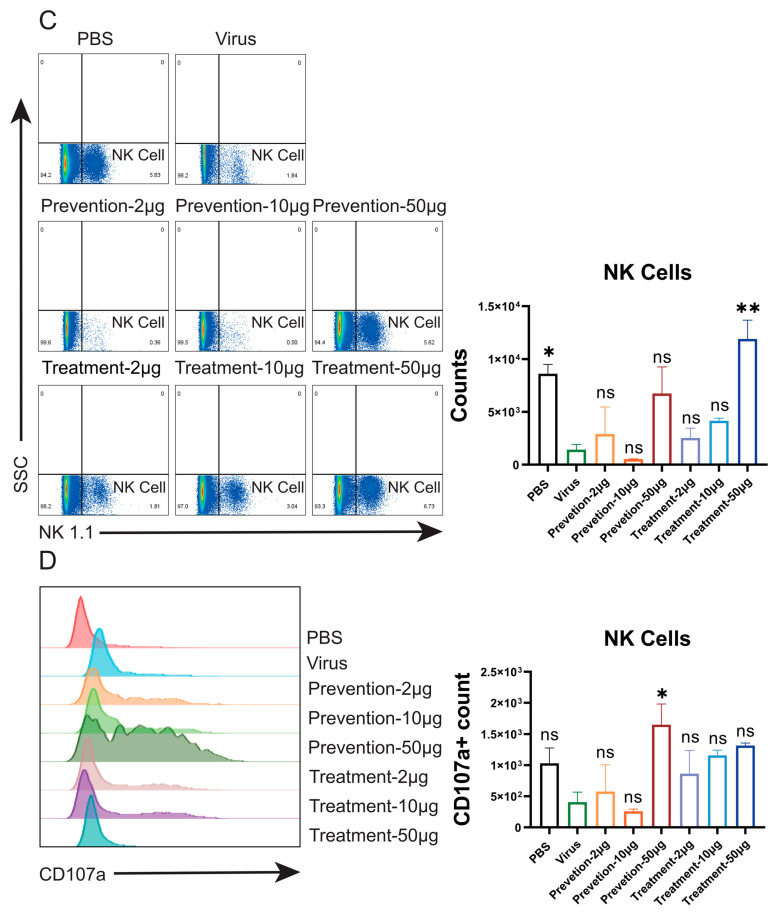

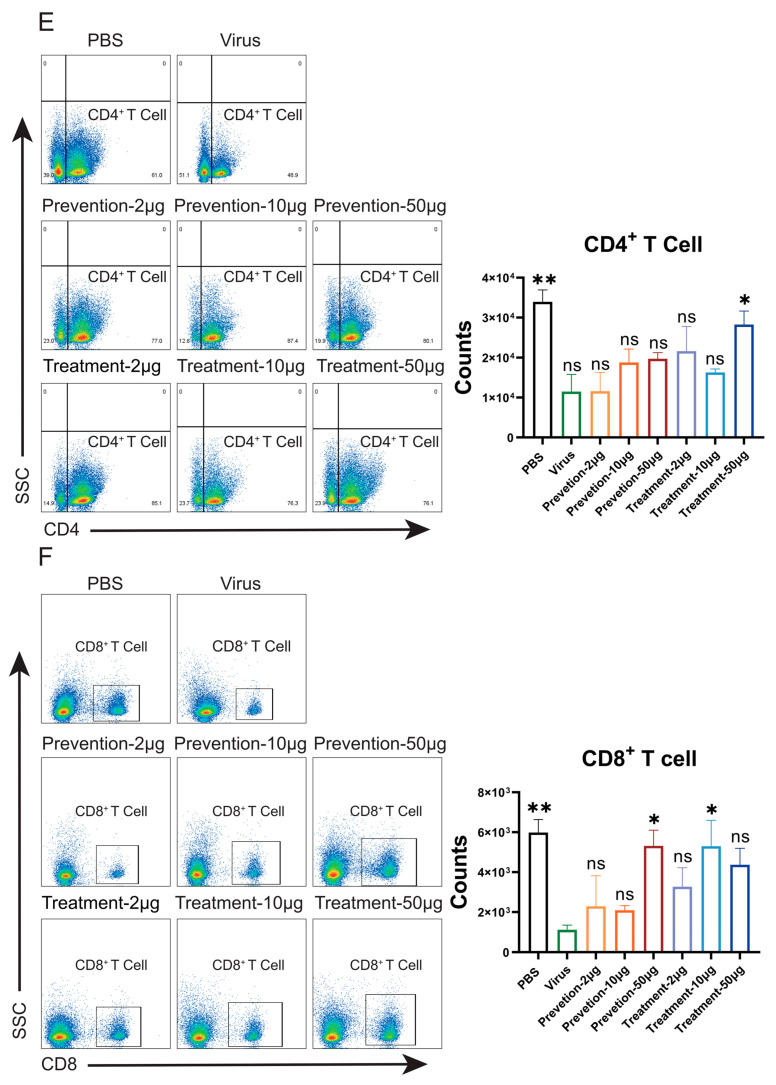
Flow cytometric analysis of immune response in STAT1^+/−^ mouse spleen. (**A**) Flow cytometric scatter plots and corresponding statistical analysis showed that the viral challenge significantly depleted dendritic cells (DCs) in the mouse spleen. Therapeutic administration at doses of 2 μg and 50 μg partially restored the DC population, albeit without reaching the levels of the PBS group. Data are presented as mean ± SD (*n* = 3). (**B**) Flow cytometric analysis and its corresponding statistical charts demonstrated that, compared to the PBS and virus-only groups, all prophylactic dosage groups and the therapeutic group at the 2 μg dose level exhibited a significant increase in the mean fluorescence intensity (MFI) of CD86^+^. Data are presented as mean ± SD (*n* = 3). (**C**) Flow cytometric scatter plots and statistical analysis demonstrated a rise in natural killer (NK) cell numbers in the 50 μg therapeutic group compared to PBS, virus, and prophylaxis groups, alongside a trend toward higher counts with escalating doses. Data are shown as mean ± SD (*n* = 3). (**D**) As shown by flow cytometric analysis and statistical charts, the 50 μg prophylactic group had significantly more CD107a^+^ cells than the PBS, virus, and therapeutic groups. Data are represented as mean ± SD (*n* = 3). (**E**) As shown by flow cytometric dot plots and statistical charts, a significant increase in CD4^+^ T cell count was restricted to the high-dose therapeutic group, in contrast to the PBS, virus, and prophylaxis groups. Data are represented as mean ± SD (*n* = 3). (**F**) As shown by flow cytometric scatter plots and corresponding statistical analysis, the virus group had decreased CD8^+^ T cell numbers compared to the PBS group, whereas conversely, an increase was seen in certain prophylactic groups (e.g., 50 μg) and the 10 μg therapeutic group. Data are represented as mean ± SD (*n* = 3). Statistical significance was determined by one-way ANOVA with Dunnett’s test (* *p* < 0.05, ** *p* < 0.01, *** *p* < 0.001, ns (not significant) *p* > 0.05).

In the STAT1^+/−^ mouse model, the candidate nanobody NVA17 demonstrated both prophylactic and therapeutic efficacy. To investigate its immunomodulatory mechanism, we analyzed downstream immune responses using flow cytometry. The results revealed that viral infection significantly compromised the quantity and function of splenic immune cells compared to the PBS control group. Specifically, post-infection, the number of dendritic cells (DCs)—key initiators of immune responses—was significantly reduced (Figure 5A), although the expression level of the surface activation marker CD86 showed no significant change (Figure 5B). Similarly, the number of natural killer (NK) cells was markedly decreased (Figure 5C), while the expression of the functional marker CD107a remained unaltered (Figure 5D). These findings suggest that viral infection may lead to a suppressed state of the innate immune system, characterized by cellular depletion and potential functional impairment. Following nanobody intervention, both the quantity and function of DCs and NK cells showed dose-dependent recovery compared with the infection group: not only were the numbers of DCs and NK cells restored (Figure 5A,C), but the expression of CD86 on DCs was also Considerably upregulated (Figure 5B). This indicates that the nanobody may neutralize the virus, thereby alleviate viral suppression of DC function and reactivate the core pathway of “antigen presentation–T cell activation.” Meanwhile, NK cell function was enhanced, as reflected by increased CD107a expression (Figure 5D), indicating effective activation of cytotoxic activity and enhanced innate immune killing. In the adaptive immune compartment, the populations of CD4^+^ helper T cells and CD8^+^ cytotoxic T cells were also restored in the treatment group compared with the virus-infected group (Figure 5E,F). This recovery, supported by the restored functions of DCs and NK cells, signifies the successful reestablishment of the “helper-killer” axis, which is essential for antigen-specific immunity.

Under antibody intervention, the functional responses of different immune cell subsets exhibited distinct kinetics. Innate immune cells, such as NK cells and DCs, were more potently activated (Figure 5A–D). It is noteworthy that prophylactic administration more effectively initiates the early activation of CD4^+^ T cells, marked by an upregulation of CD69 (Figure S3A). However, the terminal cytotoxic function (marked by CD107a) of CD8^+^ T cells was more robustly restored in the therapeutic groups (Figure S3B). This temporal hierarchy suggests that pre-existing antibodies in the prophylaxis setting provide an optimal initial signal for CD4^+^ T cell priming, leading to pronounced CD69 upregulation. Conversely, the high antigen load present during therapeutic intervention offers ample targets for CD8^+^ T cells, thereby more effectively triggering their cytotoxic program and resulting in stronger CD107a expression. Thus, the protective efficacy of antibodies extends beyond viral clearance and is critically shaped by the timing of administration, which differentially orchestrates the “helper–killer” immune axis: prophylaxis favors the initiation of adaptive immunity, whereas therapy excels at amplifying terminal cytotoxic responses.

In contrast to the robust responses of T cells and the innate immune system, flow cytometric analysis revealed a reduction in splenic B cell numbers following viral infection, a trend that was not reversed by either prophylactic or therapeutic antibody intervention (Figure S4A). Notably, no significant differences in the expression of CD38 (Figure S4B), a key B cell activation/differentiation marker, were observed across the experimental groups. Collectively, these findings indicate that the primary mechanism of the exogenous antibody therapy employed in this study is substitution rather than enhancement of the endogenous humoral response. By directly providing neutralizing antibodies, this approach efficiently clears the virus, thereby bypassing the need to activate, expand, and differentiate the host’s own B cell repertoire into antibody-secreting plasma cells. Consequently, the success of this treatment is reflected in the functional reconstitution of T cell and innate immunity, whereas the recovery of B cell numbers may depend on a longer-term regenerative process or alternative immunotherapeutic strategies.

### 3.6. Mechanism of TRIM21-Mediated Intracellular Antibody Neutralization

TRIM21 (tripartite motif-containing protein 21), a widely expressed cytoplasmic E3 ubiquitin ligase, serves as a high-affinity intracellular receptor for the Fc region of IgG and plays a critical role in innate immune responses [23]. Previous studies have demonstrated that TRIM21 recognizes the humanized monoclonal antibody 2CF4, which targets HAdV-4, through the antibody-dependent intracellular neutralization (ADIN) pathway [9]. Building on this foundation, the present study further elucidates the specific mechanism by which TRIM21 mediates ADIN. Using confocal microscopy, we first examined the intracellular colocalization of internalized nanobody NVA17 with viral particles. We then systematically evaluated the functional contribution of TRIM21 to antibody-dependent intracellular neutralization (ADIN) using engineered cell lines.

**Figure 6:**
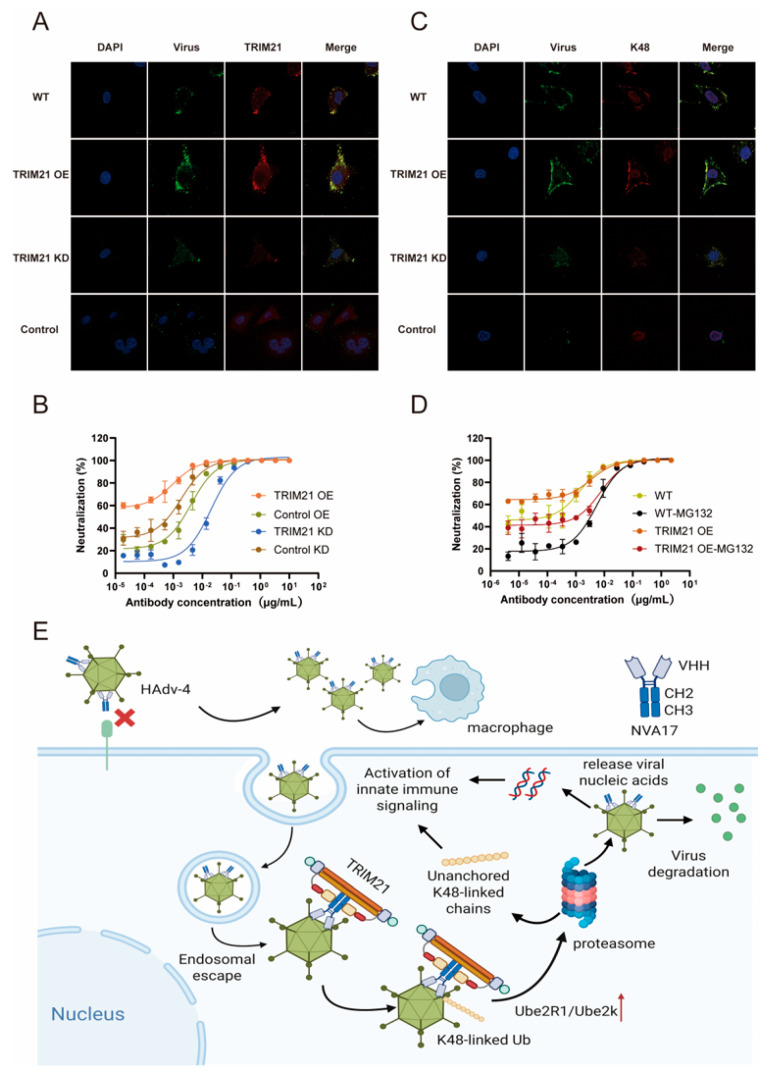
TRIM21 mediates intracellular antibody-dependent viral neutralization. (**A**) Confocal microscopy shows intracellular co-localization of nanobody-precoated HAdV-4 (green, detected with Alexa Fluor 488) and TRIM21 (red, detected with Alexa Fluor 594) in wild-type, TRIM21-overexpressing, and TRIM21-knockdown A549 cells, using a non-neutralizing nanobody as a control. (**B**) Neutralization activity of NVA17 against HAdV-4-Luc virus in A549 cells with modulated TRIM21 expression (overexpression/knockdown and controls). (**C**) Intracellular co-localization of nanobody-precoated HAdV-4 (green) and K48 ubiquitin chains (red, detected with Alexa Fluor 594) under identical cellular conditions as in (**A**). (**D**) Effect of proteasome inhibition by 1μM MG132 on NVA17-mediated neutralization of HAdV-4-Luc in wild-type versus TRIM21-overexpressing A549 cells. (**E**) NVA17 exerts dual antiviral mechanisms against HAdV-4 through extracellular neutralization and intracellular TRIM21-mediated degradation. All data are presented as the mean ± SD from three replicates in one representative experiment and are representative of three independent biological experiments.

Immunofluorescence analysis revealed that internalized NVA17 colocalized with both the virus and endogenous TRIM21 (Figure 6A). This spatial association was enhanced in TRIM21-overexpressing cells and reduced under TRIM21-knockdown conditions. Neutralization assays using Ad4-Luc virus demonstrated that TRIM21 overexpression significantly potentiated NVA17-mediated intracellular neutralization, whereas TRIM21 knockdown impaired this activity, establishing TRIM21 as a critical effector of ADIN (Figure 6B). To further dissect the underlying mechanism, we probed the involvement of K48-linked ubiquitination. Confocal imaging showed pronounced colocalization of K48-ubiquitin chains with internalized NVA17–virus complexes (Figure 6C), implicating this degradation-specific ubiquitin topology in the process. Moreover, treatment with the proteasome inhibitor MG132, which exhibited minimal cytotoxicity at working concentrations (Figure S2C,D), significantly attenuated intracellular neutralization, confirming the dependence of ADIN on a functional ubiquitin–proteasome system (Figure 6D). In summary, this study establishes TRIM21 as a central mediator of the intracellular immunity against HAdV-4 triggered by the nanobody. The mechanism involves the recognition of the internalized virus–NVA17 complex by cytosolic TRIM21, which subsequently directs the K48-linked polyubiquitination of the viral particles, thereby targeting them for proteasomal degradation (Figure 6E).

## 4. Discussion

Human adenovirus type 4 (HAdV-4) is a significant pathogen responsible for acute respiratory infections, capable of causing sporadic cases as well as outbreaks, thereby posing a threat to public health security [24]. Although vaccines against HAdV-4 are available, their use has long been restricted to military populations in the United States, effectively reducing the infection burden among recruits but remaining unavailable to civilian groups. In most regions globally, particularly in China, there is still a lack of preventive vaccines suitable for broad populations or safe and effective antiviral drugs, indicating a significant gap in control measures. Against this background, the development of novel antibody-based therapies targeting HAdV-4 is particularly urgent [25]. This study reports the development and functional validation of a panel of nanobodies targeting the hexon protein of human adenovirus type 4 (HAdV-4). The engineered candidate nanobody, NVA17, fused with a human Fc fragment, demonstrated notable therapeutic efficacy in vitro and in vivo. Our work not only identifies a potent neutralizing antibody against HAdV-4 but also comprehensively validates the downstream immune responses activated by this antibody in vivo, as well as the intracellular immune mechanisms involved—particularly underscoring the role of TRIM21 in mediating viral neutralization.

Currently, there are no reports on nanobodies targeting HAdV-4. Here, we report the isolation of high-affinity nanobodies against HAdV-4 hexon protein via sequential immunization of an alpaca. The lead candidate, NVA17, potently neutralized both Ad4-Luc recombinant virus and authentic HAdV-4 in vitro. NVA17 (IC_50_ = 4.749 ng/mL) showed enhanced potency over our previously developed fully human antibody 2CF4 (IC_50_ = 6.545 ng/mL), indicating higher affinity and therapeutic potential. Thus, NVA17 represents a promising candidate for developing new therapeutics against HAdV-4.

Phylogenetic analysis revealed a significant correlation between VHH sequences and their functional activities, with neutralizing, binding-only, and non-binding nanobodies forming distinct clusters. This genotype-phenotype association suggests that the functional divergence of nanobodies may originate from their specific germline origins and differences in complementarity-determining region (CDR) architectures. Previous studies have reported that nanobodies can target cryptic and conserved epitopes on the viral surface through their CDRs [26,27]. Our findings further support this view through epitope competition assays and structure-guided mutational analysis, indicating a diverse antibody response against the hexon protein characterized by multiple non-overlapping epitopes, with key binding residues distinct from those of the previously reported humanized monoclonal antibody 2CF4. Notably, NVA17, LUM44, and 2CF4 did not compete with each other for binding, suggesting that they recognize distinct epitopes. The unique CDR sequences of these nanobodies, particularly the structural features of HCDR3—the core antigen-binding region—provide a molecular basis for their functional differences. At the molecular level, structure prediction combined with site-directed mutagenesis identified critical residues for antigen binding. Mutations of TRP110 in NVA17 and TYR100/LEU103 in LUM44 led to a significant reduction in binding affinity, underscoring the essential role of the HCDR3 loop in antigen recognition. Particularly noteworthy, the W110A mutant of NVA17 exhibited an approximately 1000-fold increase in half-maximal effective concentration (EC_50_), confirming TRP110 as a linchpin residue responsible for its high affinity and potent neutralization activity. These findings align with recent studies on the targeting properties of nanobodies. A 2024 study by Ying Tianlei and Wu Yanling’s team demonstrated that the nanobody n425 binds to a highly conserved cryptic epitope at the G protein dimer interface. Rather than competing with the host receptor, it employs an allosteric inhibition mechanism that disrupts the tetrameric structure of the G protein, thereby blocking it is signaling to the downstream F protein [13]. That study revealed the unique epitope-targeting characteristics of nanobodies compared to conventional human-derived monoclonal antibodies. Our results corroborate these findings, as the epitope recognized by NVA17 is also distinct from known human antibodies, further supporting the unique targeting strategy of nanobodies. Moreover, NVA17 and LUM44 exhibited high specificity for HAdV-4, with no cross-reactivity to HAdV-5 or HAdV-7, indicating that they target epitopes unique to the HAdV-4 hexon protein. This characteristic provides a foundation for developing serotype-specific diagnostic and therapeutic strategies, thereby minimizing off-target effects [15]. In summary, nanobodies—through their unique CDR structures, particularly the HCDR3 region—can recognize cryptic and conserved epitopes on viral proteins, demonstrating considerable potential in countering highly mutated viral strains and providing a theoretical basis for developing next-generation antiviral strategies.

In a lethal HAdV-4 infection model using STAT1^+/−^ mice, NVA17 exhibited significant protective effects: a dose as low as 2 μg provided protection, while a 50 μg dose conferred complete resistance to the lethal challenge. Similarly, in a study on herpes simplex virus (HSV-2) conducted by Professor Zhu Shu’s team, a biparatopic nanobody they developed significantly suppressed viral replication in a lethal vaginal infection model at a dose of only 20 μg [28]. These findings collectively demonstrate the considerable potential of nanobodies in antiviral therapy across different models, highlighting the broad prospects of nanobodies as a novel antiviral strategy. Although nanobodies generally exhibit a shorter half-life in vivo compared to humanized monoclonal antibodies due to their relatively low molecular weight, often requiring frequent administration or higher doses to maintain effective blood concentrations [29], NVA17 still demonstrated notable protective activity at low doses in this study, suggesting a promising druggability profile. To further enhance its developability, improving the pharmacokinetic properties of NVA17 is of great importance. Previous studies have reported that conjugating nanobodies with serum albumin can effectively prolong their in vivo retention and improve pharmacokinetic characteristics [30], a strategy that is also applicable to the further engineering of NVA17. Prior to advancing its preclinical development, we will systematically evaluate the pharmacokinetic behavior of NVA17 to provide a basis for optimizing its half-life and designing rational dosing regimens.

Flow cytometric analysis in mouse models demonstrated that NVA17 reversed virus-induced lymphopenia and enhanced the activation and function of key immune cells. STAT1 is a central transcription factor in the interferon signaling pathway, critical for initiating innate and adaptive immunity. The extracellular neutralizing activity of antibodies can be mechanistically compromised by STAT1 deficiency, primarily due to impaired Fc-mediated effector functions. This impairment manifests as diminished ADCC and ADCP activities resulting from downregulated Fc receptor expression, coupled with functional deficits in NK cells due to maturation defects. Concurrent attenuation of IFN-γ signaling further weakens the overall immune response. Consequently, antibody efficacy evaluated in STAT1-deficient mice may substantially underestimate its true protective potential. Notably, NVA17 achieved complete protection against lethal infection in this immunocompromised STAT1^+/−^ model, unequivocally demonstrating its robust efficacy. It is crucial to emphasize that the lethal HAdV-4 infection model in STAT1^+/−^ mice better recapitulates the clinical scenario of severe adenovirus infection in immunodeficient patients, who are most susceptible to adverse outcomes. Thus, this model not only enhances the clinical relevance of our findings but also provides a more stringent and credible platform for evaluating candidate therapeutics.

Studies have demonstrated that neutralization of non-enveloped viruses involves not only extracellular antibody-mediated blockade of viral entry but also intracellular viral clearance via the TRIM21-dependent Antibody-Dependent Intracellular Neutralization (ADIN) pathway [31,32]. Given that HAdV-4 is a non-enveloped virus, we hypothesized that its specific nanobody, NVA17, may also possess ADIN activity [31]. Experimental results confirm that the antiviral activity of NVA17 is mediated not only by steric hindrance inhibiting viral entry but also significantly relies on the TRIM21-ADIN pathway. Specifically, after binding to HAdV-4 extracellularly, NVA17 is co-internalized with the virus into the cell. Within the cytoplasm, the virus-antibody complex is recognized by the E3 ubiquitin ligase TRIM21, undergoes K48-linked polyubiquitination, and is subsequently degraded by the proteasome, leading to effective viral clearance prior to replication. This indicates that NVA17 can enter cells as a virus-antibody complex and achieve intracellular viral clearance via the TRIM21-dependent proteasomal pathway, offering a novel strategy for targeting the post-entry stage of HAdV-4 infection. Notably, following the recognition of the virus-antibody complex, TRIM21 may not only activate the K48 ubiquitin-proteasome pathway but also initiate alternative ubiquitination modifications such as K63-linked ubiquitin chains, thereby triggering inflammatory signaling pathways involving NF-κB and AP-1 [33,34,35]. Furthermore, although STAT1 does not directly intersect with TRIM21 in ADIN functionality, STAT1—as a key transcription factor in the interferon signaling pathway—can regulate TRIM21 expression under immune-activated conditions [36,37]. In STAT1^+/−^ mice, the intrinsic ability of TRIM21 to initiate K48/K63 ubiquitination and activate NF-κB/AP-1 signaling remains largely intact; however, due to the absence of STAT1 signaling, the overall potency of the resulting immune protection and inflammatory response may be attenuated compared to wild-type mice. Therefore, a comprehensive elucidation of the complete intracellular ADIN mechanism elicited by NVA17 requires further validation of the activation and functional contributions of these associated inflammatory signaling pathways.

## 5. Conclusions

In conclusion, we have identified and characterized NVA17 as a highly stable, potent, and specific nanobody against HAdV-4. It confers exceptional protection in vivo through a dual mechanism: high-affinity extracellular neutralization and TRIM21-dependent intracellular degradation. Its favorable biophysical properties, efficacy in both prophylactic and therapeutic regimens, and elucidated mechanism of action position NVA17 as a promising candidate for the clinical development of anti-adenovirus therapeutics.

## Data Availability

The original contributions presented in the study are included in the article/Supplementary Materials. Further inquiries can be directed to the corresponding authors.

## Supplementary Materials

The following supporting information can be downloaded at: https://www.mdpi.com/article/10.3390/vaccines13121192/s1, Figure S1: Screening and Characterization of Nanobodies; Figure S2: Assessment of the in vivo efficacy of nanobodies and the in vitro toxicity of MG132; Figure S3: T cell activation and killing in Stat1^+/−^ mouse spleen by flow cytometry; Figure S4: T cell activation and killing in Stat1^+/-^ mouse spleen by flow cytometry.

## Abbreviations

The following abbreviations are used in this manuscript:

HAdV-4	Human adenovirus type 4
Nbs	neutralizing nanobodies
ADIN	antibody-dependent intracellular neutralization
TRIM21	tripartite motif-containing protein 21
HAdV-E	*Human mastadenovirus E*
mAb	monoclonal antibody
NK	natural killer
DMEM	Dulbecco’s Modified Eagle’s Medium
FBS	fetal bovine serum
PBMCs	peripheral blood mononuclear cells
BSA	bovine serum albumin
ELISA	Enzyme-Linked Immunosorbent Assay
SPR	Surface plasmon resonance
FBS	bovine serum
PAE	predicted aligned error
pTM	predicted template modeling
DSC	Differential Scanning Calorimetry
VH	variable heavy
CDRs	complementarity-determining regions
Tm	temperatures
Ad5-Luc	luciferase-expressing HAdV-5
Ad7-Luc	luciferase-expressing HAdV-7
DCs	dendritic cells
CDR	complementarity-determining region
