# Supplementary material for: Identification of a Highly Potent Neutralizing Nanobody Against Human Adenovirus Type 4
Source: Vaccines (Basel). 2025 Nov 25;13(12):1192. doi: 10.3390/vaccines13121192 (PMC12737500; doi:10.3390/vaccines13121192)
Supplement: Supplementary file 1 [file vaccines-13-01192-s001.zip › vaccines-3967335-supplementary.pdf]

Identification of a Highly Potent Neutralizing Nanobody Against Human Adenovirus Type 4

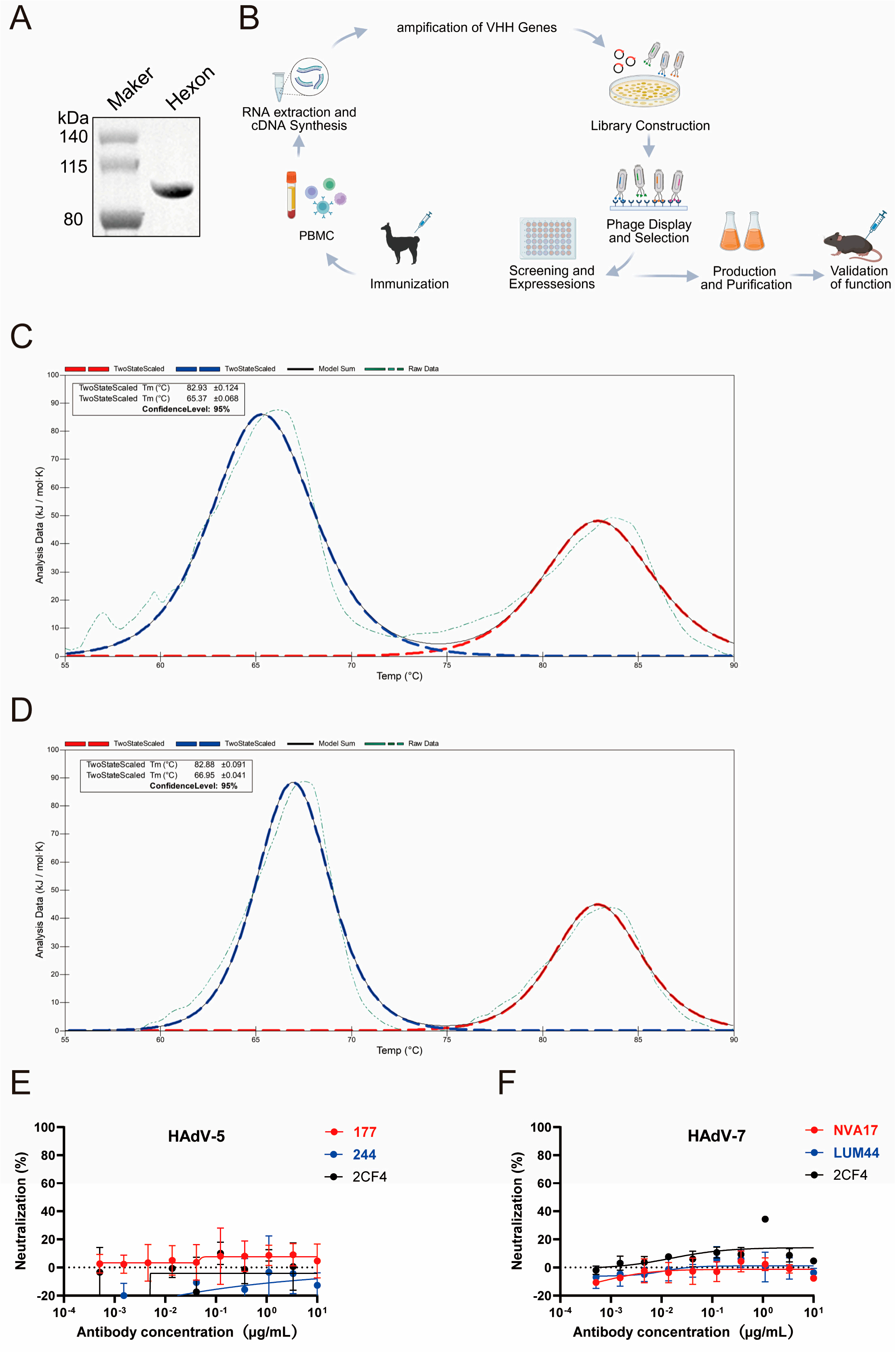

Figure S1. (A) Western blot analysis of purified HAdV-4 Hexon protein. (B) Schematic of the nanobody isolation and planning process. (C) Differential scanning calorimetry (DSC) thermogram of NVA17 fitted to a two-state scaled model, revealing two transition peaks (T_{m1} = 82.93 ± 0.124 °C, T_{m2} = 65.37 ± 0.068 °C).

Curves represent original data (green), total model sum (blue), and the fitted curve (red). (D) DSC thermogram of LUM44 analyzed under the same model, showing two thermal transitions (T_{m1} = 82.88 ± 0.091 °C, T_{m2} = 65.95 ± 0.041 °C). (E) Neutralization activity of NVA17 against Ad5-Luc virus in A549 cells. Data are presented as mean ± SD from one representative experiment. (F) Neutralization activity of NVA17 against Ad7-Luc virus in A549 cells.

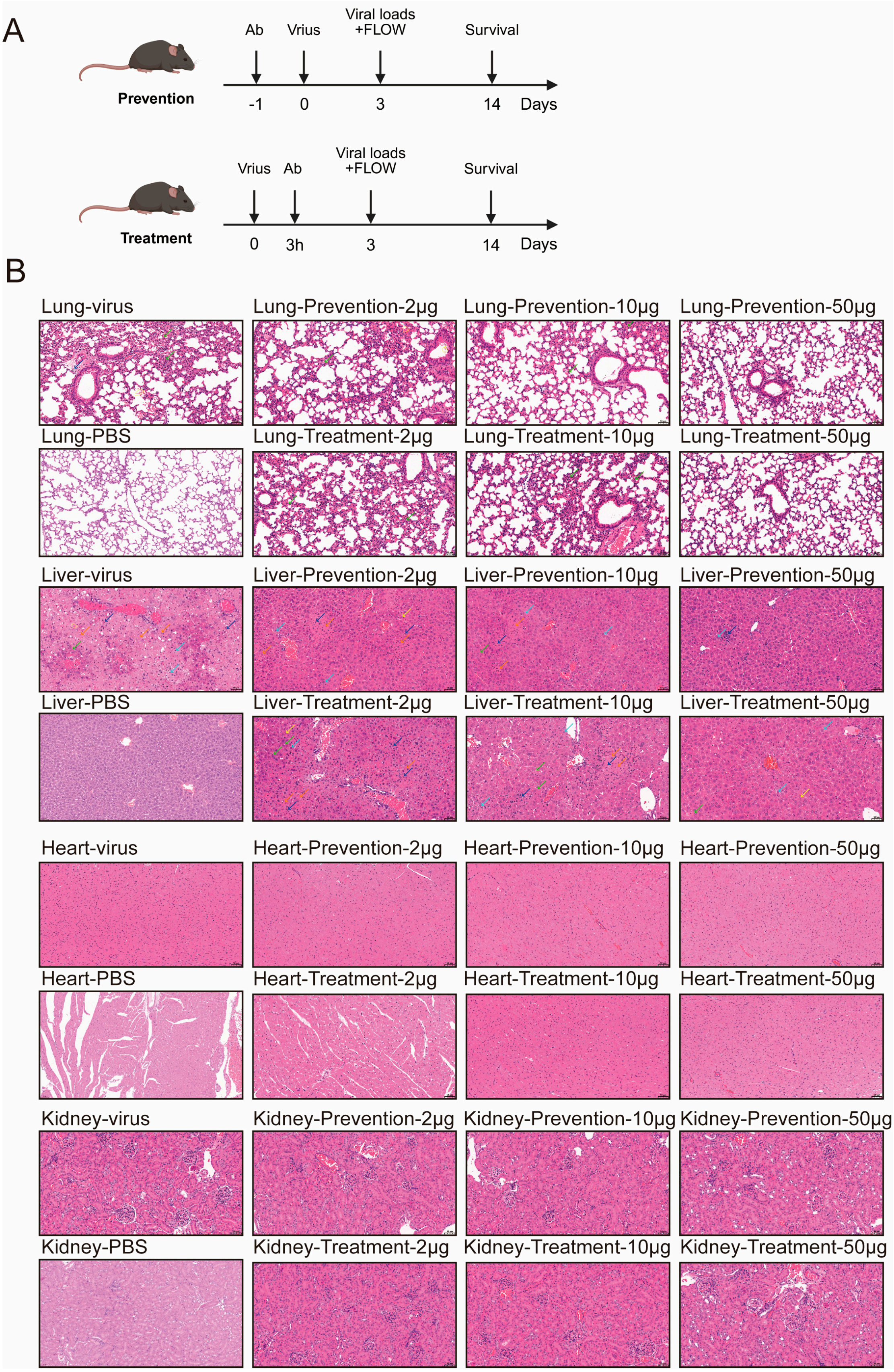
A
Prevention
Ab
Vrius
Viral loads
+FLOW
Survival
-1
0
3
14
Days
Treatment
Vrius
Ab
Viral loads
+FLOW
Survival
0
3h
3
14
Days
B
Lung-virus
Lung-Prevention-2μg
Lung-Prevention-10μg
Lung-Prevention-50μg
Lung-PBS
Lung-Treatment-2μg
Lung-Treatment-10μg
Lung-Treatment-50μg
Liver-virus
Liver-Prevention-2μg
Liver-Prevention-10μg
Liver-Prevention-50μg
Liver-PBS
Liver-Treatment-2μg
Liver-Treatment-10μg
Liver-Treatment-50μg
Heart-virus
Heart-Prevention-2μg
Heart-Prevention-10μg
Heart-Prevention-50μg
Heart-PBS
Heart-Treatment-2μg
Heart-Treatment-10μg
Heart-Treatment-50μg
Kidney-virus
Kidney-Prevention-2μg
Kidney-Prevention-10μg
Kidney-Prevention-50μg
Kidney-PBS
Kidney-Treatment-2μg
Kidney-Treatment-10μg
Kidney-Treatment-50μg

13

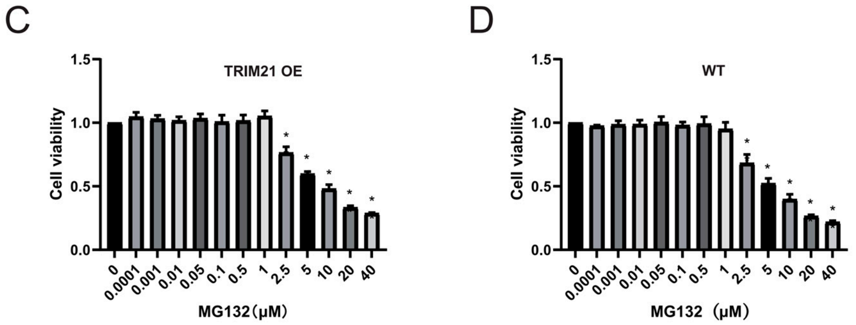

Figure S2. (A) Schematic of the immunization schedule for STAT1+/- mice. (B) Pathological sections of lung, liver, heart, and kidney tissues from STAT1+/- mice at 3 days post-infection. In the virus-infected group, lung tissues exhibited alveolar wall thickening, inflammatory cell infiltration (green arrows), necrotic debris (bule arrows), and hemorrhage, with occasional congestion (yellow polygons). Compared to this group, the low-dose group showed reduced alveolar wall thickening, attenuated inflammation in half of the samples, and less hemorrhage. The medium-dose group had decreased thickening and inflammatory infiltration in most samples, increased congestion, but no significant hemorrhage. The high-dose group presented reduced thickening, congestion, and inflammatory infiltration, with no other notable changes. In liver tissues of the virus-infected group, common histopathological findings included hepatocellular edema (cyan arrows), chromatin margination (yellow arrows), focal fatty degeneration (green arrows), moderate to severe necrosis (orange arrows), mild inflammatory infiltration (blue arrows), and hemorrhage (purple dashed polygons). Compared to this group, all treatment groups showed reduced necrosis extent; both low- and medium-dose groups exhibited decreased proportions of samples with fatty degeneration and edema, whereas the high-dose group demonstrated reduced fatty degeneration and congestion. No significant pathological alterations were observed in cardiac or renal tissues across all experimental groups. (C) Viability of wild-type A549 cells after 24 h treatment with increasing concentrations of MG132 (**$P < 0.01$). (D) Viability of TRIM21-overexpressing A549 cells after 24-hour treatment with MG132 across the same concentration range.

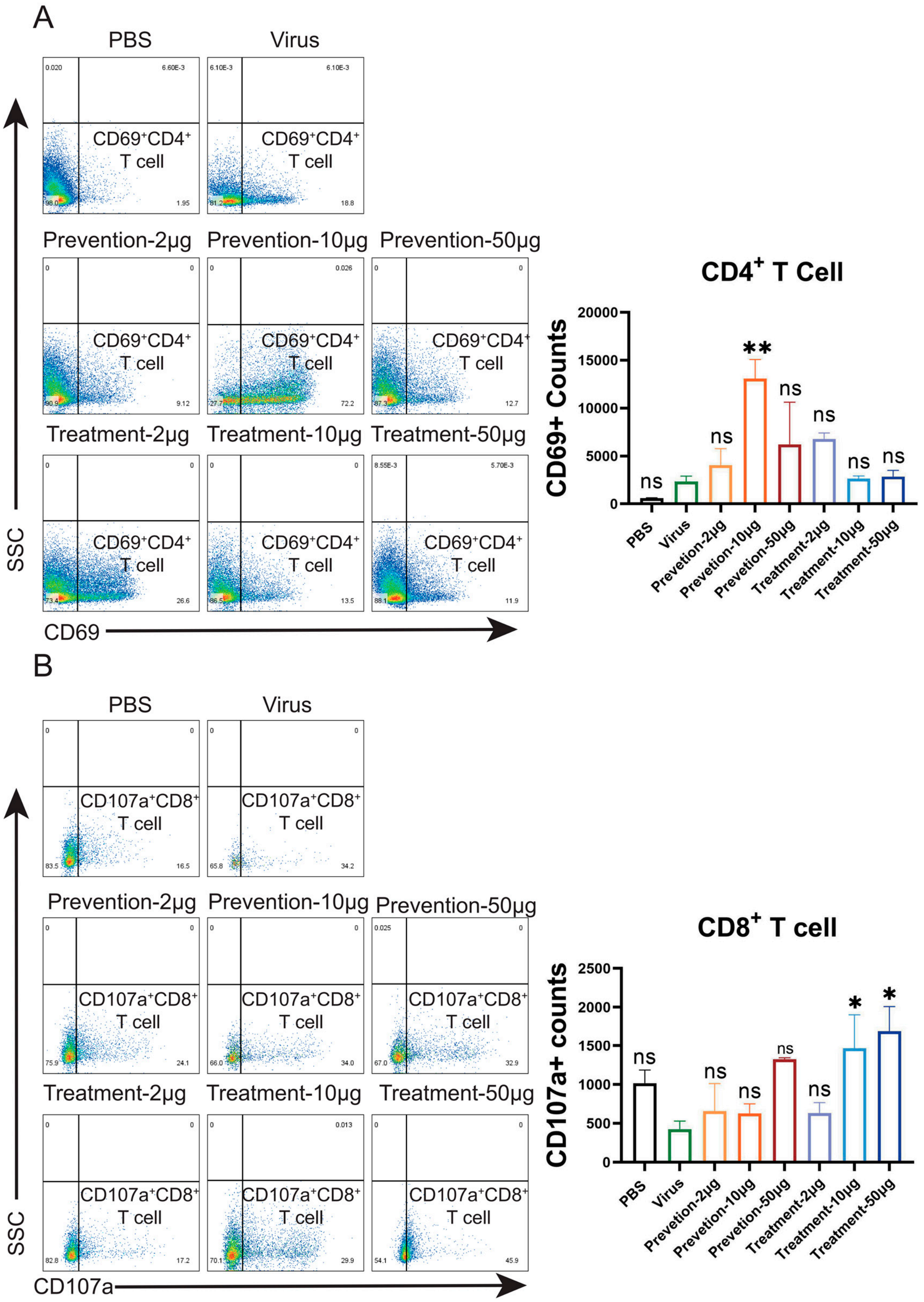

Figure S3. T cell activation and killing in Stat1+/- mouse spleen by flow cytometry. (A) Flow cytometric dot plots and corresponding statistical analysis revealed that the count of CD69⁺CD4⁺ T cells was increased in the Prevention 10μg group relative to the PBS and Virus controls. The remaining groups did not differ significantly. Values represent mean ± SD (n = 3) (B) Flow cytometric scatter plots and statistical analysis indicated a modest increase in CD107a⁺ cytotoxic T cells (CTLs) in the 10μg and 50μg therapeutic groups relative to the PBS and virus controls, whereas no significant changes were detected in any other

groups. Values represent mean ± SD (n = 3) Statistical significance was determined by one-way ANOVA with Dunnett's test (*p < 0.05, **p < 0.01).

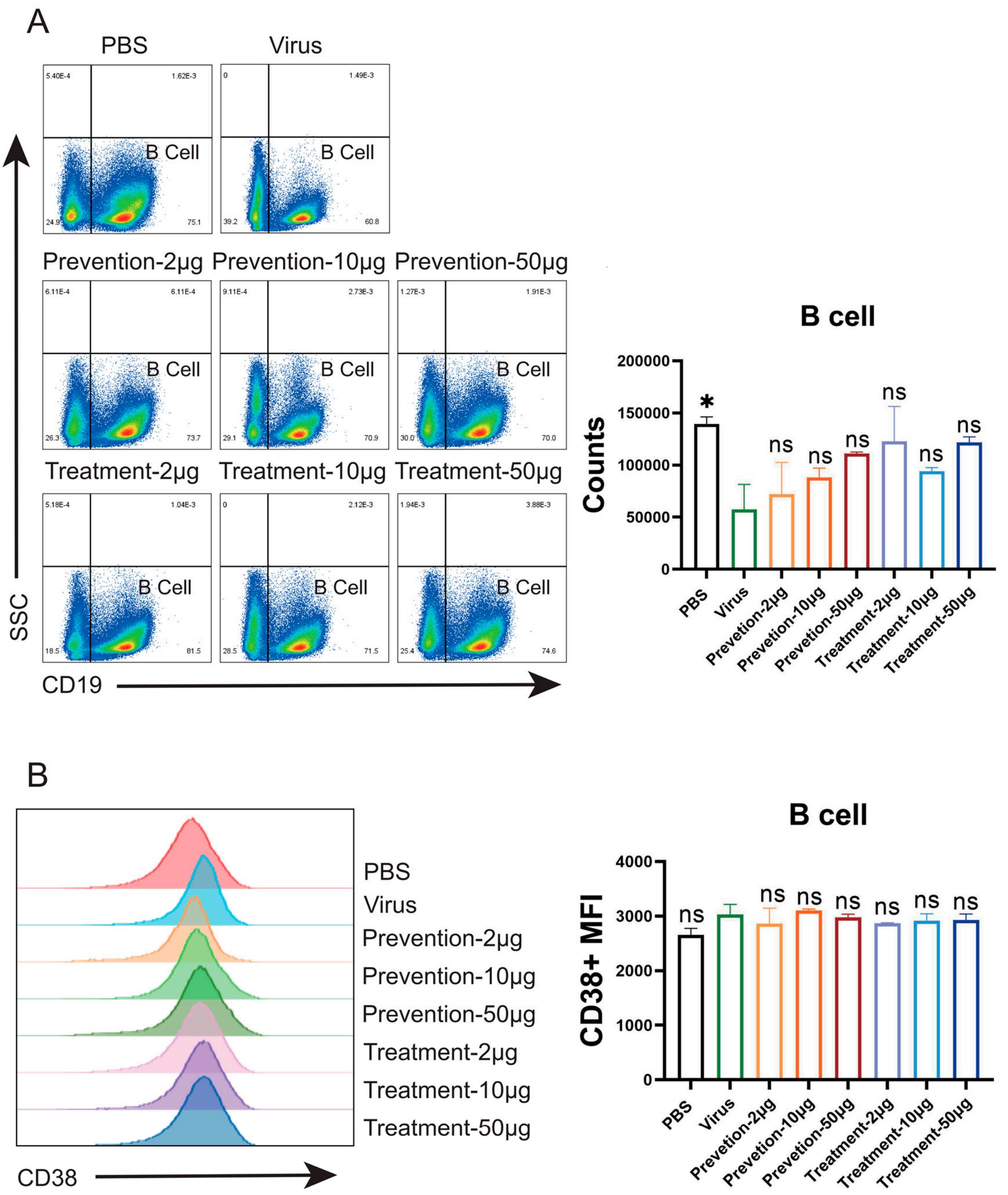

Figure S4. T cell activation and killing in Stat1+/- mouse spleen by flow cytometry. (A) Flow cytometric dot plots and corresponding statistical analysis revealed that Whereas the Virus group exhibited a significant reduction in B cell numbers compared to the PBS group, all Prevention and Treatment groups showed no significant difference from the PBS controls. Values represent mean ± SD (n = 3). (B) Flow cytometric analysis and its statistical plots showed that the CD38⁺ MFI of B cells were comparable across

all treatment groups, including PBS, Virus, and the different doses of Prevention and Treatment. Values represent mean ± SD (n = 3), Statistical significance was determined by one-way ANOVA with Dunnett's test (*p < 0.05, **p < 0.01).
